# Identifying Oncogenic Missense Single Nucleotide Polymorphisms in Human *SAT1* Gene Using Computational Algorithms and Molecular Dynamics Tools

**DOI:** 10.1002/cnr2.2130

**Published:** 2024-07-23

**Authors:** Md. Mozibullah, Marina Khatun, Md. Asaduzzaman Sikder, Mohammod Johirul Islam, Mehbuba Sharmin

**Affiliations:** ^1^ Department of Biochemistry and Molecular Biology Mawlana Bhashani Science and Technology University Tangail Bangladesh

**Keywords:** in silico, missense SNPs, molecular dynamics simulations, *SAT1* gene

## Abstract

**Background:**

The human *SAT1* gene encodes spermidine/spermine N1‐acetyltransferase 1 (SSAT1), a regulatory biological catalyst of polyamine catabolism. Numerous essential biological processes, such as cellular proliferation, differentiation, and survival, depend on polyamines like spermidine and spermine. Thus, SSAT1 is involved in key cellular activities such as proliferation and survival of cells and mediates various diseases including cancer. A plethora of studies established the involvement of missense single nucleotide polymorphisms (SNPs) in numerous pathological conditions due to their ability to adversely affect the structure and subsequent function of the protein.

**Aims:**

To date, an in silico study to identify the pathogenic missense SNPs of the human *SAT1* gene has not been accomplished yet. This study aimed to filter the missense SNPs that were functionally detrimental and pathogenic.

**Methods and Results:**

The rs757435207 (I21N) was ascertained to be the most deleterious and pathogenic by all algorithmic tools. Stability and evolutionary conservation analysis tools also stated that I21N variant decreased the stability and was located in the highly conserved residue. Molecular dynamics simulation revealed that I21N caused substantial alterations in the conformational stability and dynamics of the SSAT1 protein. Consequently, the I21N variant could disrupt the native functional roles of the SSAT1 enzyme.

**Conclusion:**

Therefore, the I21N variant was identified and concluded to be an oncogenic missense variant of the human *SAT1* gene. Overall, the findings of this study would be a great directory of future experimental research to develop personalized medicine.

## 1 Introduction

Polyamines, multifunctional polycations such as spermidine and spermine, are crucially important for various fundamental biological processes including cellular proliferation, differentiation, and survival [1, 2]. In physiological conditions, polyamines exhibit a protonated nature. As a result, they can interact with nucleic acids and certain types of proteins to regulate DNA and protein synthesis as well as fundamental cellular function [3]. Deregulated polyamine levels have been entailed in a variety of cancers [4], cystic fibrosis [5], and Alzheimer's disease [6]. Increased levels of polyamines are essential to mediate transformation and progression of tumor [7]. Therefore, polyamine levels are tightly regulated by synthesis, degradation, import, and export mechanisms [8].

The human *SAT1* gene is located at the Xp22.1 chromosomal position having six exons and five introns, which encodes a 171 amino acids containing spermidine/spermine N^1^‐acetyltransferase 1 (SSAT1) protein [9]. An indispensable enzyme for lowering the levels of polyamine is SSAT1, which catalyzes N^1^‐acetylation of polyamine, specifically spermidine and spermine for the conversion of less charged products acetylated spermidine and spermine for exporting from the cell or degrading the acetylated tagged polyamines by the acetyl polyamine oxidase [10]. SSAT1 protein regulates cell migration through the interaction with integrin α9β1, for instance, leukocyte migration to the site of inflammation [11]. *SSA*T1 also has been reported to be interacted with HIF1α (a transcription regulator of genes, which involve in glucose metabolism, angiogenesis, cellular stress, and apoptosis) [12, 13], SLC3A2 (a diamine transporter for exporting acetylated polyamine) [14], and eIF5A (SSAT1‐mediated acetylation of eIF5A controls translation) [15].

Approximately 90% genetic variability of the human genome is caused by single nucleotide polymorphisms (SNPs) [16]. SNPs that are situated in the coding region and alter the encoding amino acid are called missense variants, which can adversely affect the native function of the corresponding protein. Missense variants are responsible for numerous pathological conditions including cancer and may also persuade the disease susceptibility and treatment [17]. Therefore, distinguishing and identifying disease‐causing missense variants from a large pool of neutral variants is a widely and crucially studied area. In vivo experimental studies for all missense SNPs of a gene are hard to perform, time‐consuming, and costly, hence researchers gravitate to ascertain and curtail missense SNP for ultimate in vivo examination. Therefore, the in silico study has been a widely used and renowned scheme to specify disorder related missense SNPs precisely from a plethora of neutral missense variants [18]. Based on the in silico approach, various studies have efficaciously identified disease‐associated missense SNPs [19].

The NCBI dbSNP database enlisted a significant number of missense variants for the human *SAT1* gene. Even though missense variations are linked to diseases, no computational research has been conducted to assess these enlisted missense SNPs of the human *SAT1* gene to determine disease‐causing missense mutations such as oncogenic mutations out of neutral variants. Thus, rational computational studies of the human *SAT1* gene would be a great way to curtail the number of missense variants and filter the pathogenic variants from neutral missense SNPs with minimal time and less resources, labor, and cost for experimental study.

In this study, a wide range of computational algorithmic tools were utilized to assess the functional impacts of all dbSNP database enlisted missense SNPs and identified the pathogenic missense variants. The molecular dynamics simulations (MDSs) were executed by mimicking the physiological conditions to elucidate the structural impacts of the selected missense SNPs on the human *SAT1* encoding SSAT1 protein.

## 2 Methods

### 2.1 Data Mining

The UniProtKB database [20] was exploited to retrieve the general information and fasta sequence of SSAT1 protein. The NCBI‐dbSNP database deposits the SNPs with various information on human genes [21]. This database was utilized to obtain all deposited SNPs of the human *SAT1* gene and particularly missense SNPs‐related data including SNP IDs, genomic, and protein coordinates were transferred to a Microsoft Excel sheet for detailed study. The three‐dimensional shape of SSAT1 protein (PDB ID: 2G3T [22]) was retrieved from the Protein Data Bank [23].

### 2.2 Functional Consequences and Pathogenicity Analysis of the Missense Variants

A wide range of algorithmic tools that operate on different methodologies were employed to predict the functional impacts and disease‐associated missense variants with high accuracy and reliability [18, 24]. All the employed algorithmic tools in our study were divided into two categories that are functional impact and pathogenicity prediction tools.

#### 2.2.1 Functional Consequences Analysis of the Missense Variants

Functional consequences of all missense SNPs of the human *SAT1* gene were assessed by the PROVEAN [25], FATHMM [26], and SNAP‐2 [27] algorithmic tools.

PROVEAN works on the principle of sequence homology‐based methods to generate a specific score for predicting the functional impact of a missense SNP with sensitivity, specificity, and accuracy of 78.85%, 79.55%, and 79.20%, respectively. This tool takes primary protein sequence and amino acid variant to search homologous sequences by BLAST and represents a PROVEAN score. Score ≤–2.5 is predicted to have a “deleterious” effect on the protein function [25].

With excellent sensitivity, specificity, and accuracy of 86%, FATHMM (trained method) utilizes a species‐independent approach to classify the functionally deleterious and neutral variants. FATHMM tool can be utilized to identify the genetic mutations that cause phenotypic differences, for example, pathogenic phenotype. In our study, unweighted algorithm was employed to find out the functionally damaging variants. Prediction score of ≥−3.0 for a variant denotes detrimental effect on that protein's function [26].

SNAP‐2, a neural network tool, efficiently predicts the effect of a missense SNP with 86% sensitivity, 75% specificity, and 83% accuracy. Generally, SNAP2 performs noticeably better than other techniques. Prediction is based on the evolutionary data that are generated from the multiple sequence alignments. Prediction score ranging from −100 to +100, which indicates strong neutral and non‐neutral prediction, respectively [27].

#### 2.2.2 Pathogenicity Analysis of the Missense Variants

Pathogenic or diseased missense SNPs were predicted by the SuSPect [28], VEST‐4 [29], and SNPs&GO [30].

SuSPect outperforms other algorithmic tools in predicting disease‐associated missense variants with 75% sensitivity and 82% accuracy by integrating the features of network‐level and sequence conservation. UniProtKB ID and amino acid variants are acceptable input format. The outcome scores spanning from 0 to 100, where value of ≥50 implies diseased/pathogenic variant [28].

VEST‐4, a supervised machine‐learning tool, which was utilized for prioritizing rare missense SNPs with the likelihood of disease causing in human. VEST performs better than some of the most widely used techniques for evaluating and curtailing missense variants based on their propensity of causing diseases. Carter et al. demonstrated that the area under curve (AUC) for the VEST performance and precision‐recall curves were both 0.92, demonstrating the high sensitivity and specificity of the VEST tool in classifying missense mutations that have functional impacts for corresponding protein activity [29].

SNPs&GO, a support vector machine algorithm, exploits the functional annotation of a protein to predict if the missense mutation is disease related or neutral, which has 83% sensitivity, 80% specificity, and 82% accuracy. SNPs&GO accumulates information in a distinctive framework that incorporates protein sequence, evolutionary data, and action as expressed in the terms used in the Gene Ontology [30].

### 2.3 Protein Stability Analysis

Missense SNPs can alter the structural features of the corresponding protein, including stability. I‐Mutant3.0 [31] and SDM [32] were utilized to assess the alteration in the SSAT1 protein stability due to the missense variant.

To forecast the impact of nsSNPs in coding areas, the I‐Mutant3.0 uses a variety of machine learning algorithms by analyzing two aspects, for example, stability of protein folding and loss of functionality [31]. The input parameters for I‐Mutant3.0 were SSAT1 fasta sequence, wild‐type, and variant residues position, temperature 37°C, and pH 7.4.

SDM uses the frequencies of amino‐acid alteration in the homologous families of protein to generate a score of stability based on the differences on the free energy among the variant and wild‐type protein [32]. The PDB ID: 2G3T, mutation, and chain A were the input formats for the SDM tool.

### 2.4 Evolutionary Conservation Analysis

The ConSurf web server determines the conservation score for all residues of a protein from 1 to 9 to unveil the conservation profile throughout the evolution via the method of Bayesian computation. The ConSurf also identifies the functional and structural amino acid residues with information on the exposed and buried residues in the three‐dimensional structure of the corresponding protein [33, 34]. The PDB ID: 2G3T and chain A were submitted with default parameters to determine the outcomes of the ConSurf server.

### 2.5 Interacted Protein–Protein Network of SSAT1 Protein by STRING v11.5 Database

Protein–protein interactions are crucial in maintaining the native functions of the particular protein and the corresponding homeostasis of the functional system. Therefore, STRING v11.5 was employed to determine the interacting partners of SSAT1 protein with a score of high confidence [35].

### 2.6 Molecular Dynamics Simulation

The retrieved three‐dimensional x‐ray crystallographic structure of SSAT1 (PDB ID: 2G3T) was processed by removing water molecules, ligands, and ions in the Discovery Studio 2020. The Swiss‐PDB Viewer (SPDV) [36] was utilized to eliminate bad contacts and add atoms that are missing in the structure, subsequently subjected to energy minimization with the GROMOS96 force. Later, the I21N variant was also generated and subjected to energy minimization by the same tool. One of the most renowned free and open‐source packages of software for dynamical simulations is GROMACS. It offers a wide range of calculating tools, analysis, and preparation [37]. To elucidate the alteration in the dynamic nature of the protein due to the selected I21N variant, molecular dynamics (MD) simulations were executed by GROMACS package [37] for a period of 100 ns with CHARM27 force field [38] in the server of WebGRO for macromolecular simulations (https://simlab.uams.edu/) [38]. This tool, which has made by the University of Arkansas for Medical Sciences (UAMS), provides free public service for worldwide researchers. The input parameters for solvation and neutralization of the system were a simple point‐charge (SPC) water model [39] in the Triclinic box and 0.15 M NaCl salt. The steepest descent for 5000 steps was utilized for energy minimization. The simulation system was equilibrated by using the NVT/NPT at 310 K temperature and 1 bar pressure. Leap‐frog algorithm was selected as the MD integrator and the time for simulation was 100 ns. For trajectory analysis, 1000 frames for each simulation of wild‐type and mutant protein were generated. The trajectory files were analyzed to reveal the root mean square deviation (RMSD), radius of gyration (Rg), solvent accessible surface area (SASA), root mean square fluctuation (RMSF), and number of H‐bonds.

### 2.7 Analysis and Presentation of Data

Tables and figures were generated to present the outcomes of our comprehensive in silico investigations. The retrieved data and outcomes of the study were analyzed and presented (in terms of tables and figures) by utilizing MS words, Excel, PowerPoint, IBM SPSS v25, and GraphPad Prism v8.

## 3 Result

**FIGURE 1:**
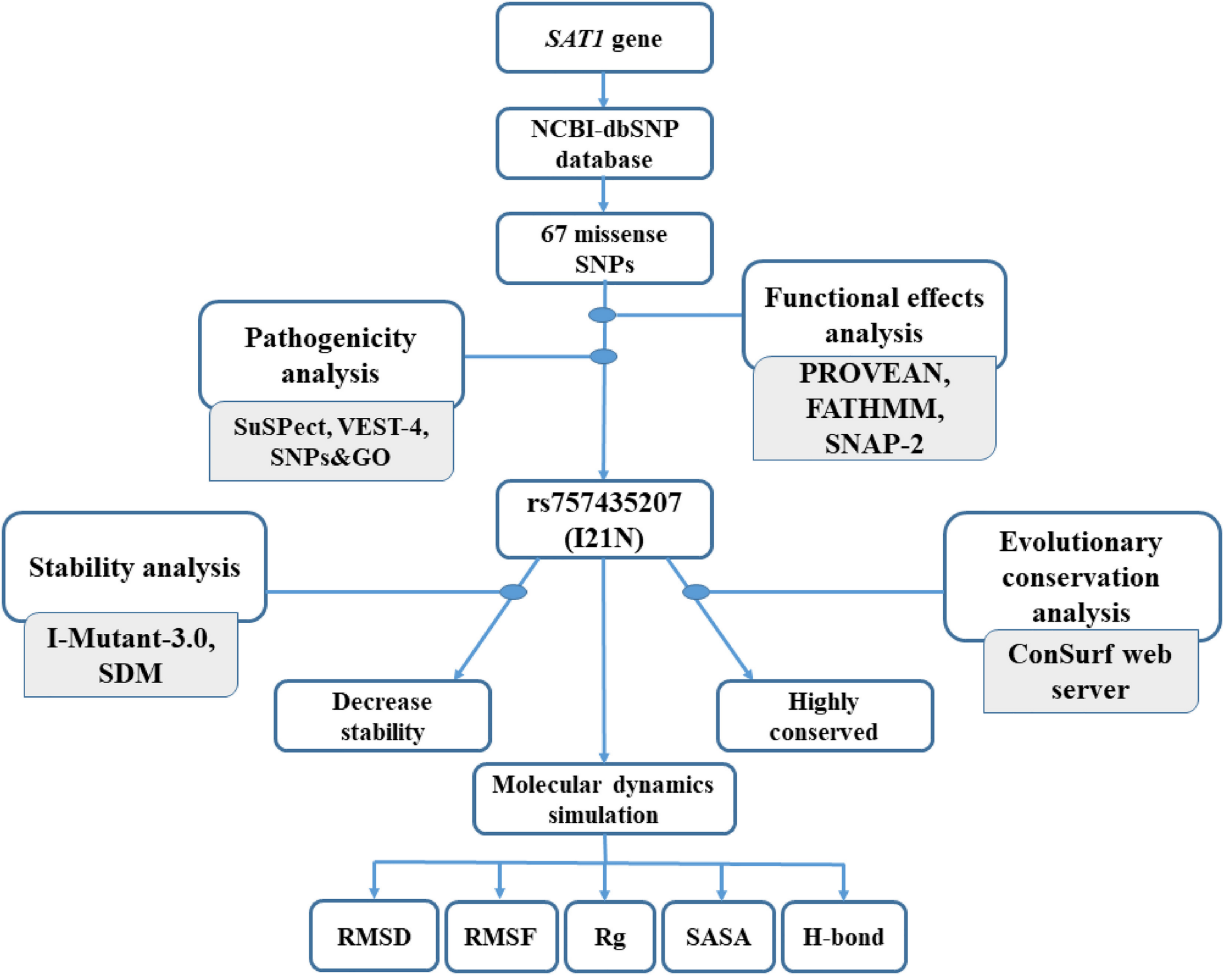
Flowchart representation of methodology. Retrieved missense SNPs from the NCBI‐dbSNP database were subjected to functional effects and pathogenicity analysis, followed by the stability and evolutionary conservation analysis. The structural effects of the selected variants on the SSAT protein were then evaluated by the molecular dynamics simulation.

The flowchart demonstrates the overall methodology of the identification of pathogenic missense SNPs along with the stability, evolutionary conservation, and MD simulation analysis to characterize the detailed impacts of the identified pathogenic variants on the SSAT1 protein (Figure 1).

### 3.1 SNPs Data

**FIGURE 2:**
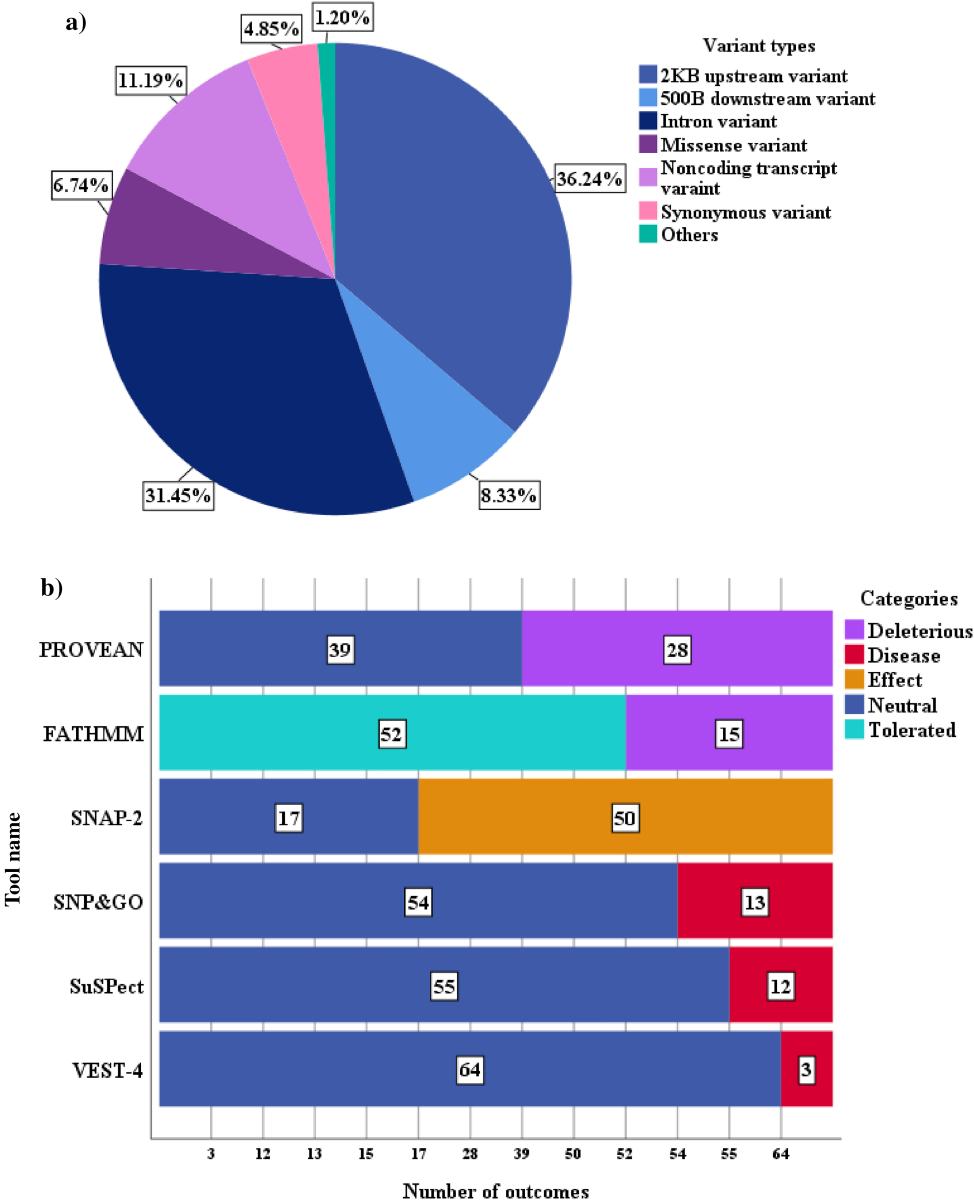
SNP‐associated information in human *SAT1* gene. (a) The pie chart illustrates the total SNP layout with variant types from the dbSNP database. (b) The stacked bar chart displays the entire outcomes of the employed algorithmic tools.

The NCBI‐dbSNP database deposited 1752 variants of the human *SAT1* gene (Table S1). Out of all variants, the highest number of variants was the 2KB upstream variant (36.24%), followed by the intron variant (31.45%), noncoding transcript variant (11.19%), 500B downstream variant (8.33%), missense variant (6.74%), and other variants including frameshift, splice donor, initiator codon variant, and so forth (Figure 2a).

### 3.2 Identification of Functionally Damaging and Pathogenic Missense Variants

The NCBI‐dbSNP database enlisting missense variants of the human *SAT1* gene were retrieved and analyzed to determine the functionally deleterious and disease‐causing missense variants by six algorithmic tools (Table S2) such as FATHMM, PROVEAN, SNAP‐2, SuSPect, VEST‐4, and SNPs&GO (Table 1).

**TABLE 1:** Functional effects and pathogenicity analysis of all retrieved missense variants of the human *SAT1* gene.

No.	SNP ID	A.A change	PROVEAN	FATHMM	SNAP‐2	SuSPect	VEST‐4 (*p*)	SNPs&GO
1	rs11404	R121S	Neutral	Tolerated	Effect	19	0.23206	Neutral
2	rs11404	R121C	Deleterious	Damaging	Effect	8	0.20464	Neutral
3	rs1139915	M30R	Deleterious	Tolerated	Effect	16	0.07307	Neutral
4	rs1804460	L89F	Deleterious	Tolerated	Effect	43	0.16253	Neutral
5	rs2855495	K26E	Neutral	Tolerated	Effect	8	0.21536	Neutral
6	rs139915283	A12D	Neutral	Tolerated	Effect	10	0.59366	Neutral
7	rs145594541	I35V	Neutral	Tolerated	Neutral	10	0.90426	Neutral
8	rs145980431	A12T	Neutral	Tolerated	Neutral	18	0.48801	Neutral
9	rs145980431	A12S	Neutral	Tolerated	Neutral	14	0.79496	Neutral
10	rs147672674	E131G	Neutral	Tolerated	Effect	19	0.46554	Neutral
11	rs148483557	D99H	Neutral	Damaging	Effect	20	0.2353	Neutral
12	rs747031398	K39N	Neutral	Tolerated	Neutral	12	0.23753	Neutral
13	rs747031398	K39N	Neutral	Tolerated	Neutral	52	0.23753	Neutral
14	rs748119306	V96M	Deleterious	Damaging	Effect	52	0.46554	Neutral
15	rs751600110	D13N	Deleterious	Damaging	Effect	77	0.10859	Disease
16	rs752149094	E108D	Neutral	Tolerated	Effect	20	0.30442	Neutral
17^a^	**rs757435207**	**I21N**	**Deleterious**	Damaging	Effect	**54**	**0.00961**	**Disease**
18	rs757435207	I21T	Deleterious	Damaging	Effect	60	0.01893	Neutral
19	rs757797199	G68E	Deleterious	Tolerated	Effect	17	0.17852	Disease
20	rs761372150	T10A	Neutral	Tolerated	Effect	30	0.78767	Neutral
21	rs765638178	I71T	Neutral	Damaging	Effect	30	0.04838	Neutral
22	rs768921302	I6T	Deleterious	Damaging	Effect	83	0.06679	Neutral
23	rs769555216	M97I	Neutral	Tolerated	Effect	12	0.23672	Neutral
24	rs771632138	E62G	Deleterious	Tolerated	Effect	13	0.56512	Neutral
25	rs774822224	H63N	Neutral	Tolerated	Effect	19	0.53922	Neutral
26	rs777012184	R7P	Deleterious	Damaging	Effect	86	0.17529	Disease
27	rs778776707	A57T	Deleterious	Damaging	Neutral	56	0.94029	Disease
28	rs780634321	F94L	Deleterious	Tolerated	Effect	35	0.62635	Disease
29	rs868263851	Y29H	Neutral	Tolerated	Effect	8	0.38235	Neutral
30	rs868263851	Y29D	Neutral	Tolerated	Effect	9	0.5212	Neutral
31	rs933833989	Y78C	Deleterious	Damaging	Effect	72	0.21506	Disease
32	rs950111257	F127C	Deleterious	Tolerated	Effect	65	0.22073	Disease
33	rs1014715667	G104D	Deleterious	Damaging	Effect	77	0.16425	Disease
34	rs1016338251	D40Y	Deleterious	Tolerated	Effect	22	0.12276	Neutral
35	rs1016484612	F46L	Deleterious	Tolerated	Effect	43	0.40684	Disease
36	rs1166003456	M118V	Neutral	Tolerated	Effect	15	0.69487	Neutral
37	rs1189367982	C54S	Deleterious	Tolerated	Effect	43	0.37557	Disease
38	rs1190623283	S124G	Neutral	Tolerated	Effect	19	0.3446	Neutral
39	rs1204204970	P66R	Neutral	Tolerated	Effect	15	0.39976	Neutral
40	rs1229945436	D147V	Deleterious	Tolerated	Effect	19	0.66684	Disease
41	rs1263893758	D160N	Neutral	Tolerated	Effect	14	0.67038	Neutral
42	rs1269688315	K166I	Deleterious	Damaging	Effect	30	0.38235	Neutral
43	rs1301130726	N138T	Neutral	Tolerated	Neutral	12	0.28094	Neutral
44	rs1322842368	S123G	Neutral	Tolerated	Effect	26	0.2518	Neutral
45	rs1328342274	I35M	Neutral	Tolerated	Neutral	12	0.86287	Neutral
46	rs1363595764	R121H	Neutral	Tolerated	Effect	13	0.22336	Neutral
47	rs1419531675	M125T	Deleterious	Tolerated	Effect	63	0.29724	Neutral
48	rs1427582120	Q33E	Neutral	Tolerated	Neutral	12	0.28216	Neutral
49	rs1444826638	K111N	Deleterious	Tolerated	Neutral	22	0.22923	Neutral
50	rs1484283875	Y52H	Deleterious	Tolerated	Effect	35	0.70792	Neutral
51	rs1601856931	R119K	Neutral	Tolerated	Effect	5	0.32142	Neutral
52	rs1601857029	T169A	Neutral	Tolerated	Neutral	3	0.67574	Neutral
53	rs1922571078	K3E	Neutral	Tolerated	Neutral	7	0.39227	Neutral
54	rs1922571273	V5M	Neutral	Tolerated	Neutral	15	0.6468	Neutral
55	rs1922571273	V5L	Neutral	Tolerated	Neutral	20	0.84921	Neutral
56	rs1922572327	P8S	Deleterious	Tolerated	Effect	25	0.56857	Neutral
57	rs1922595347	M30V	Neutral	Tolerated	Neutral	20	0.17549	Neutral
58	rs1922605182	L42V	Neutral	Tolerated	Neutral	16	0.96873	Neutral
59	rs1922607943	K61Q	Neutral	Tolerated	Neutral	12	0.64447	Neutral
60	rs1922608736	W64R	Neutral	Tolerated	Effect	16	0.25271	Neutral
61	rs1922677412	S70R	Neutral	Tolerated	Effect	9	0.2104	Neutral
62	rs1922678596	P83L	Deleterious	Tolerated	Effect	27	0.08592	Disease
63	rs1922685087	F103C	Neutral	Tolerated	Effect	22	0.36403	Neutral
64	rs1922685397	I105T	Deleterious	Damaging	Effect	29	0.24593	Neutral
65	rs1922685556	S107L	Deleterious	Damaging	Effect	44	0.32972	Neutral
66	rs1922686134	N112Y	Neutral	Tolerated	Effect	23	0.63172	Neutral
67	rs1922697582	E171K	Neutral	Tolerated	Effect	6	0.45633	Neutral

^a^ Variant that was predicted to be functionally deleterious and pathogenic by all tools is highlighted in the row by the bold mark.

#### 3.2.1 Identification of Functionally Damaging Variants

At first, FATHMM, a species‐independent based approach, was utilized to identify the functionally damaging missense variants of the human *SAT1* gene. Out of 67 missense mutations, FATHMM (unweighted) algorithm predicted that 15 missense variants crossed the threshold value (≤−3.0) and were classified as damaging variants. PROVEAN (≤−2.5), a widely accepted tool of functional effect analysis, was also employed in our study, which identified 28 variants as functionally damaging as they violate the threshold value of neutral variants. Subsequently, SNAP‐2 (>0), an improved version of SNAP, distinguished 50 variants that affect protein function from 17 neutral variants. Figure 2b displays the detailed outcomes of the functional analysis.

#### 3.2.2 Identification of Pathogenic Variants

To further confirm the previous analysis, we also performed the pathogenicity analysis of all missense variants of the human *SAT1* gene. VEST‐4, SuSPect, and SNPs&GO algorithmic tools were assigned to predict the disease‐associated/pathogenic missense variants. VEST‐4 classified the lowest number of missense variants as pathogenic (three pathogenic variants) by assigning a *p* value of less than 0.05. The SuSPect (≥50) and SNPs&GO (>0.5) identified almost the same number of disease‐associated missense variants that were 12 and 13, respectively, while the rest of the variants didn't violate the threshold value and classified as neutral variants (Figure 2b).

### 3.3 Selecting the Most Damaging and Pathogenic Variant

The results received from the functional and pathogenicity analysis were compared and combined to determine the most harmful and pathogenic missense variants. One missense variant with an SNP ID of rs757435207 (I21N) was predicted as functionally deleterious to the SSAT1 protein and pathogenic by all employed functional and pathogenicity analysis algorithmic tools. Hence, this variant was selected for further investigation.

### 3.4 Protein Stability Analysis

Structural impact in terms of stability of the SSAT1 protein due to the I21N variant was assessed by the I‐Mutant3.0 and SDM, which are protein stability prediction tools upon missense mutation. Both I‐Mutant3.0 and SDM predicted DDG values of −1.77 and −0.86 Kcal/mol, respectively, suggesting the substantial reduction of the SSAT1 protein's stability due to the I21N variant (Table 2).

**TABLE 2:** Stability analysis of the SSAT1 protein due to the I21N variant.

SNP ID	Variant	I‐Mutant3.0		SDM	
		Stability	DDG value (Kcal/mol)	Stability	DDG value (Kcal/mol)
rs757435207	I21N	Decrease	−1.77	Reduce	−0.86

### 3.5 Evolutionary Conservation Analysis

**FIGURE 3:**
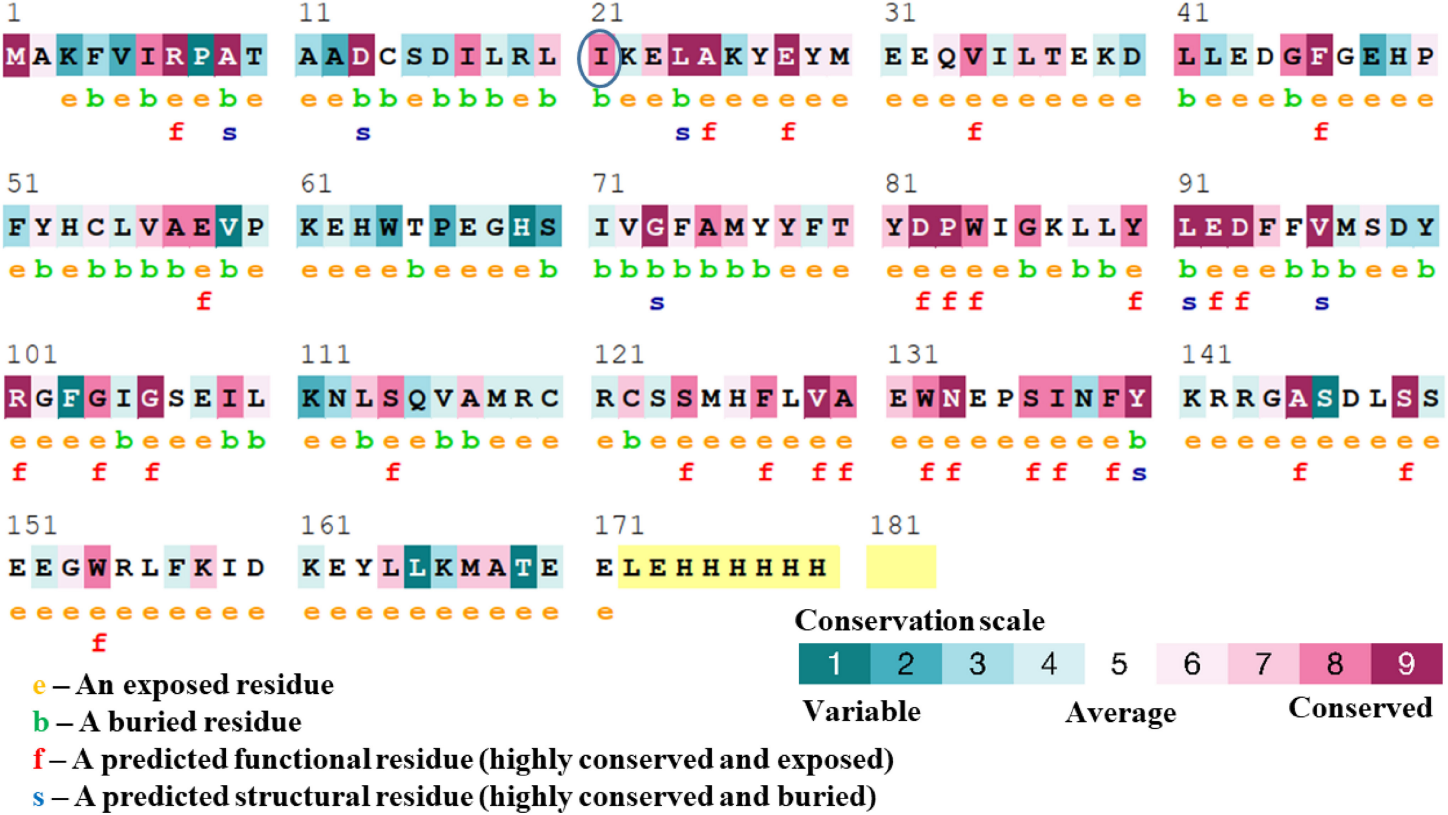
The ConSurf server generated the conservation patterns of SSAT1 protein throughout the evolution. Residue is colored based on the conservation score.

Evolutionary conserved residue of a protein is essential in maintaining the native structure as well as the function of that protein. Hence, we analyzed the conservation profile of the SSAT1 protein to reveal the conservation pattern throughout the evolution by ConSurf server. The ConSurf assigned a score of 8 for the wild‐type residue of the I21N variant, indicating the highly conserved scenario throughout the evolution (Figure 3). The ConSurf also suggested that I21 is a buried residue.

### 3.6 Interacted Protein–Protein Network of SSAT1 Protein by STRING

**FIGURE 4:**
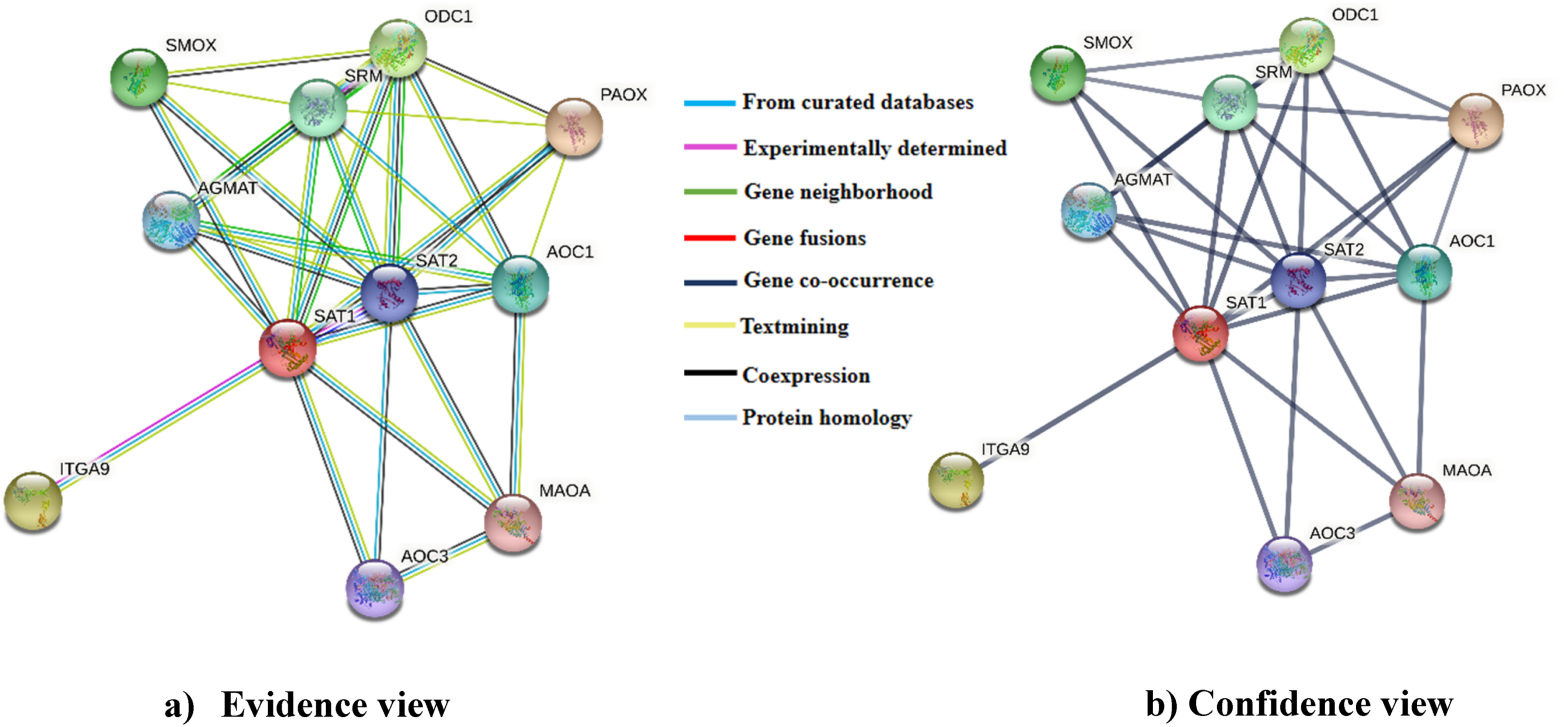
Protein interaction partners of SSAT1 protein determined by the STRING database with high confidence (0.700). (a) In the evidence view, the line color illustrates the type of interaction. (b) In the confidence view, the line thickness demonstrates the strength of interaction.

Interacted network of SSAT1 protein was determined by the STRING server, which suggested that SSAT1 protein has functional interactions with a variety of proteins involved in maintaining polyamine homeostasis (synthesis and degradation), lymphocyte extravasation, cell migration, and so forth. STRING identified a network of protein interactions with ITGA9, AOC3, MAOA, SAT1, AOC1, PAOX, ODC1, SRM, AGMAT, and SMOX (Figure 4). The I21N variant‐mediated alteration in the SSAT1 protein may hamper native interactions with these proteins and associated functions.

### 3.7 Generation of Mutant 3D Structure of the SSAT1 Protein

**FIGURE 5:**
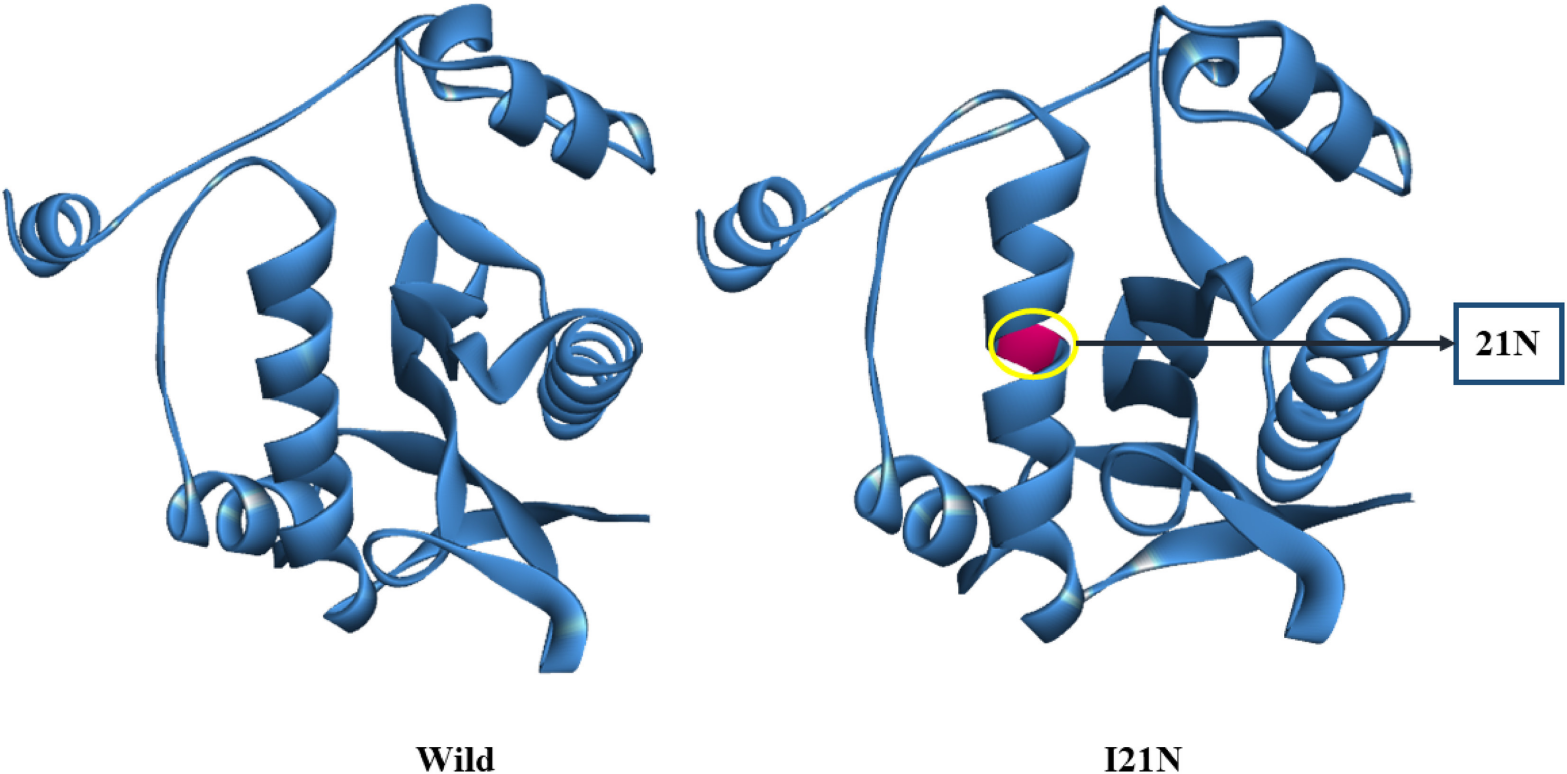
The 3D structure of the wild‐type and I21N variant of the SSAT1 protein.

I21N variant's three‐dimensional (3D) structure was created using the Discovery Studio 2020 and Swiss‐PDV Viewer [36]. The 3D structure of the wild‐type and I21N variant were displayed in Figure 5.

### 3.8 Molecular Dynamics Simulation

To unveil the impact of the I21N variant on the structural stability and dynamic nature of SSAT1 protein, MDS were accomplished in physiological conditions for 100 ns time course. The RMSD, RMSF, Rg, SASA, and the number of H‐bonds were analyzed for both wild‐type SSAT1 protein and I21N variant.

#### 3.8.1 RMSD Analysis

**FIGURE 6:**
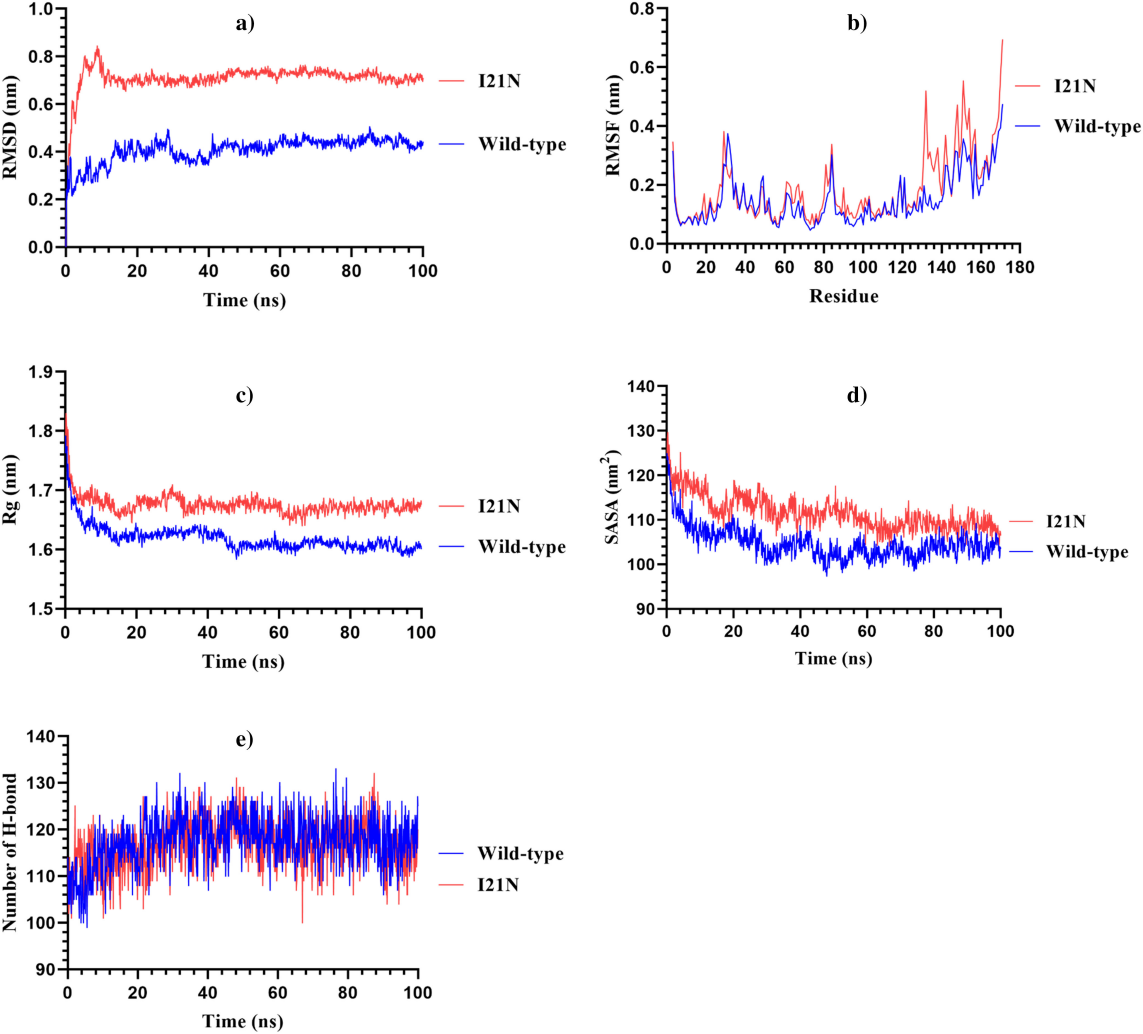
Analysis of the effects of I21N variant in SSAT1 protein throughout the 100 ns MD simulation time by determining the (a) The root mean square deviation (RMSD), (b), root mean square fluctuation (RMSF), (c) radius of gyration (Rg), (d) solvent accessible surface area (SASA), and (e) number of H‐bonds. The blue and red colors represent the wild‐type and I21N variant.

In order to reveal the conformational stability of the wild‐type and I21N variant during the simulation period, RMSD was analyzed. RMSD analysis denoted that wild‐type SSAT1 protein attained an equilibration state after 25 ns simulation time, while I21N variants equilibrated after 8 ns, after that specific time, maintained the equilibrated state for the rest of the simulation time. It was evident from the total RMSD analysis that the I21N variant showed higher RMSD than the wild‐type SSAT1 during the entire simulation (Figure 6a). Calculated average RMSD value (Table 3) for wild‐type and I21N was 0.406 and 0.709 nm, respectively, indicating that the I21N variant caused substantially more deviated conformational stability than the wild‐type protein.

**TABLE 3:** Average data of RMSD, RMSF, Rg, SASA, and H‐bond throughout the molecular dynamics simulation of wild‐type and mutant of SSAT1.

Protein	RMSD (nm)	RMSF (nm)	Rg (nm)	SASA (nm^2^)	H‐bond in number
Wild‐type	0.406	0.149	1.619	104.665	∼118
I21N variant	0.709	0.184	1.676	111.611	∼116

Abbreviations: Rg, radius of gyration; RMSD, root mean square deviation; RMSF, root mean square fluctuation; SASA, solvent accessible surface area.

#### 3.8.2 RMSF Analysis

Root mean square fluctuation analysis was accomplished to determine the dynamic behavior of each residue of the wild‐type and I21N variant. The wild‐type and variant showed different fluctuating nature of their residues (Figure 6b). The highest fluctuation of the variant proteins occurred in the residues from Ala130 to Arg143 and Ala145 to Phe157. Most of the residues of the I21N variant showed higher fluctuations than the wild‐type SSAT1 protein. The average RMSF values for wild‐type and variant were 0.149 and 0.184 nm, respectively (Table 3). Therefore, the I21N mutation notably changed the flexibility of the SSAT1 protein. Alteration in the dynamic conformation of these residues in the I21N variant protein could adversely affect the native activity of the SSAT1 enzyme.

#### 3.8.3 Rg Analysis

Radius of gyration is an MD parameter that illustrates the overall structural compactness and stability of the simulated protein systems. Rg analysis revealed that the I21N variant introduced a notable deviation in the compactness of the SSAT1 protein structure (Figure 6c). The I21N variant showed a sharp increase in the Rg value (1.682 nm) between 20 and 35 ns, while the wild‐type retained a steady Rg value (1.626 nm). An increased average Rg value (Table 3) was observed for the I21N (1.676 nm) compared to the wild‐type SSAT1 protein (1.619 nm). Overall, the higher average Rg value of the I21N variant indicated the introduction of variant‐mediated higher flexibility and lower compactness than the wild‐type SSAT1 protein, which was consistent with the RMSD and RMSF outcomes.

#### 3.8.4 SASA Analysis

Solvent accessible surface area measures the accessible surface area of a protein to the solvent during the simulation time. Higher and lower SASA values indicate an alteration in the structural conformation due to the expansion (that means the protein is unfolded) and compact state, respectively. SASA analysis clearly indicated that compared to the wild‐type, a substantial difference in the structural conformation was attained in the I21N variant protein (Figure 6d). The average SASA value of the I21N variant (111.611 nm^2^) was higher than the wild‐type protein (104.665 nm^2^) (Table 3). Therefore, it can be definitely said that the I21N variant adversely affects the structural conformation inducing substantial protein expansion and unfolding. The SASA results supported the outcomes of RMSD, RMSF, and Rg.

#### 3.8.5 H‐Bond Analysis

The number of H‐bonds during the simulation period was calculated (Figure 6e), which showed that the total number of H‐bonds was higher in the wild‐type SSAT1 protein with a calculated average value of ∼118 than the I21N variant with a calculated average value of ∼116 (Table 3) indicating the alteration in the protein stability.

Overall, the higher RMSD value and lower number of intramolecular H‐bonds stated that the I21N mutation introduced substantial destabilizing effects on the native SSAT1 structure. In addition, the SSAT1 protein structure exhibited structural distortion due to the overall expansion and unfolding consequences of the I21N mutation, as demonstrated by the higher values of RMSF, Rg, and SASA. Therefore, the I21N variant may cause an alteration in the native enzymatic and functional activity, which could lead to a pathogenic/oncogenic state.

## 4 Possible Oncogenic Mechanism of I21N Mutated SSAT1 Protein

As polyamines involve fundamental cellular processes including cellular proliferation, survival, and so forth, it has been evident that SSAT‐induced polyamine depletion dramatically reduced cancer cell migration, invasion, and proliferation via the AKT/GSK3β/β‐catenin signaling pathway [40]. Recent investigations have also suggested a potential action of intracellular polyamines in the expression and activation of MAPK kinase pathway and growth‐associated *c‐fos* and *c‐myc* proto‐oncogenes for cell proliferation [41].

**FIGURE 7:**
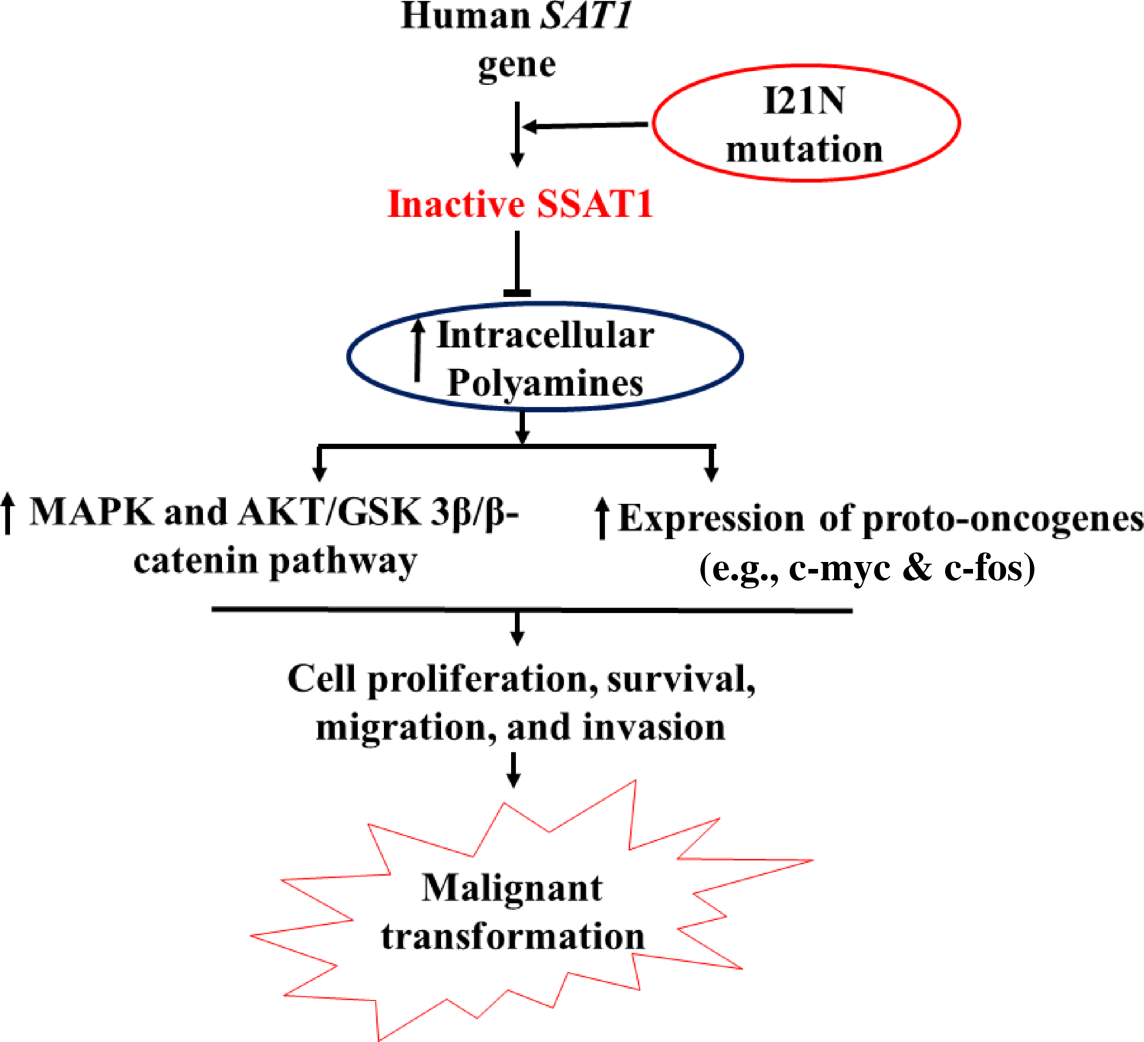
Possible oncogenic mechanism of the I21N mutated SSAT1 protein. The I21N mutation may cause catabolic dysfunction of the SSAT1 protein increasing intracellular polyamines level. Therefore, elevated polyamines convert the cells to a malignant state due to uncontrolled expression and activation of corresponding pathways and proto‐oncogenes.

Our study indicated that I21N mutation could hamper the catabolic function of the SSAT1 enzyme causing elevated intracellular polyamines level. Therefore, elevated polyamines could lead to the malignant transformation of cells through the uncontrolled expression and activation of the aforementioned pathways and proto‐oncogenes (Figure 7).

## 5 Discussion

The deep sequencing technology has made it possible to identify a plethora of missense SNPs that are increasingly deposited in the SNPs depository database. Very few portions of identified missense SNPs are pathogenic [42, 43]. So the biggest challenge is to distinguish the pathogenic missense variant from a large number of neutral variants by existing experimental approaches that are very costly and labor‐intensive procedures [42]. This drawback has been overcome by the development of numerous algorithmic prediction tools considering their various advantages compared to experimental approaches. The *SAT1* encoding SSAT1 protein is a regulatory enzyme of polyamine homeostasis that is involved in fundamental cellular activities including cell proliferation and survival. SSAT1 has also been reported to be associated with various diseases including cancer [1, 2, 17, 44, 45, 46]. In silico approach of identifying the pathogenic missense SNPs is not performed yet. A combinatorial selection of multiple tools that work based on different methodologies can improve the accuracy and validity of the prediction [18]. In this study, six algorithmic tools (PROVEAN, FATHMM, SNAP‐2, SuSPect, VEST‐4, and SNPs&GO) based on different methodologies were utilized to identify the functionally damaging and disease‐associated missense SNPs. Out of 67 missense SNPs, only I21N (rs757435207) was predicted to be deleterious and pathogenic by all functional and pathogenic missense variant predictors of computational tools.

Missense mutation not only affects the structure of the corresponding protein but also its stability. Therefore, protein stability prediction due to missense mutations gives an idea of the molecular basis of disease [47]. Stability prediction tools (I‐Mutant‐3.0 and SDM) predicted that the I21N variant would decrease the stability of the SSAT1 protein. Evolutionary conserved residues are crucial in maintaining the native structure and function of the protein. Consequently, substitution of a highly conserved residue leads to the pathogenic state [48]. The ConSurf server stated that wild‐type residue of the I21N variant is highly conserved throughout the evolution. Therefore, the I21N variant can alter the protein structure, consequently adversely affecting the native function of the SSAT1 protein. Proteins are interacted with their functional partners to mediate the assigned cellular processes. Hence, revealing the functions of a protein requires identifying the interacted partners [49]. We identified the interacting network of SSAT1 proteins with other partner proteins by STRING database, which unveiled that SSAT1 interacts with a number of proteins that are involved in the cell migration and homeostasis of polyamine through the maintenance of polyamine metabolism. Out identified pathogenic variant (I21N) may alter the functional interactions with other proteins of polyamine metabolism, leading to the development of diseases that generally develop due to the alteration of normal polyamine metabolism such as cancers [4], Alzheimer's disease [6], and so forth. The functional features including catalytic reactions, signal transduction, and interactions of a protein are determined by the rigid structures and dynamic behaviors [50]. To gain detailed insights into the deleterious effects of the I21N variant in terms of conformational dynamics and stability, MDS were executed. RMSD analysis revealed that the I21N variant causes a less stable protein structure. RMSF, Rg, SASA, and H‐bond analysis confirmed the higher residual fluctuation, flexibility, expansion, unfolding, and loss of stability. The outcomes of the MDS were strongly consistent with the prediction of algorithmic tools.

Polyamines are involved in cellular growth, stability of the chromatin structure, synthesis of proteins and nucleic acids, apoptosis, differentiation, and preventive action of oxidative damage, and so forth. Polyamines are generally maintained in millimolar amounts in mammalian cells, indicating tight regulation of polyamines through the pathways of metabolism, export, and import [51, 52]. Deregulated levels of polyamines have been substantiated in cancerous cells, more specifically, a high level of polyamines is needed in the transformation and progression of tumors. SSAT1 is a regulatory enzyme in reducing polyamine levels via excreting and degrading spermine and spermidine. Thus, there is a direct connection between SSAT1 activity and the efflux of the polyamine from the mammalian cells [7, 53].

Missense mutations that alter protein stability, conformational dynamics, and network of H‐bonds compared to the relevant characteristics of the wild‐type protein can have a damaging effect on the protein function. Functional alteration of a protein and pathological conditions are interconnected [17]. Therefore, all computational algorithmic tools and MDS suggested that I21N is a pathogenic missense variant that has substantially deleterious effects on the SSAT1 protein and disturbs its polyamine catabolic function. Therefore, elevated polyamines cause the cells to be formed in a malignant state due to uncontrolled expression and activation of MAPK and AKT/GSK 3β/β‐catenin pathways and *c‐fos* and *c‐myc* proto‐oncogenes (Figure 7). HIF1α is a transcription regulator of genes involving glucose metabolism, angiogenesis, cellular stress, and apoptosis [12]. Hypoxia is a condition that supports tumor growth and progression having an inadequate supply of oxygen through the activity of HIF1α [54]. In hypoxia conditions, SSAT1 regulates the HIF1α activity. The binding of SSAT1 to the HIF1α triggers ubiquitin‐mediated degradation of that HIF1α in low oxygen conditions by preserving the interaction of HIF1α and RACK1 [12]. Therefore, cellular inability to inactivate the activity of HIF1α fosters the development of cancer. Disturbance due to I21N‐mediated structural modification in the binding capacity between SSAT1 and HIF1α could also lead to pathogenic state, for example, cancer. SLC3A2, a functional interaction partner of SSAT1, is a transporter responsible for polyamine efflux. In addition, the efflux mechanism is crucial in maintaining the homeostasis of polyamine. SLC3A2‐mediated polyamine efflux is coupled with arginine influx. It is evident that SSAT1 and SLC3A2 are co‐localized indicating the role of their interaction in efflux mechanism [13, 14]. So SSAT1 structural alteration due to I21N variant could lead to hamper their interaction, subsequently the loss of acetylated polyamine's efflux. Deregulated levels of polyamine could lead to pathogenic state, for example, cancer [13, 14]. It is suggested that eIF5A triggers apoptosis through the regulation of p53 protein expression [55]. eIF5A contains hypusine residue, which on one side mimics the polyamine, that is, essential for the activity of eIF5A. At the cellular level, hypusine acetylation negatively controls the eIF5A activity. SSAT1 enzyme does not treat free hypusine residue as a substrate suggesting potential interaction between SSAT1 and eIF5A for acetylation [56]. Furthermore, it is also showed that eIF5A can lose its functionality when acetylation is carried out by the SSAT1 protein [56]. So inactivated and structurally distorted SSAT1 due to I21N mutation may not interact with eIF5A and confer immortality leading to cancerous cells. Numerous biological processes such as inflammation, tissue repair and regeneration, and embryogenesis depend on cell migration [57]. Migrating cells expresses the integrin α9β1, for instance, that mediate the migration of leukocytes to the area of injury and chronic inflammation. It has also been reported that SSAT1 also has functional interaction with integrin α9β1 to regulate the leukocytes migration [11]. Structural change in the SSAT1 protein due to I21N variant could also hamper migration of the immune cells in tissue injury and inflammation that opens a window of extensive experimental approaches.

Therefore, we speculate that the I21N variant could hamper the polyamine reduction function of the SSAT1 protein and functional interaction with HIF1α, SLC3A2 and eIF5A that may lead to develop a diseased state such as a cancerous condition. With the concept of targeted therapies, cancer treatment has undergone a substantial change. Gene mutation surveillance of patients is going to be considered a promising tool for the diagnosis, categorization, and therapy of malignancies in terms of personalized medicine [58, 59]. Therefore, our identified oncogenic mutation could be helpful in assessing the resistance and effectiveness to targeted treatments, subsequently allowing the development of personalized healthcare facilities [58].

This extensive computational study enlightens the identification of I21N oncogenic missense mutation I21N of the human *SAT1* gene, while no experimental approach has been conducted to confirm the oncogenic properties. All employed computational tools work on the principle of algorithms that are trained on a range of disease‐related missense mutations and controls [28, 29, 60]. Therefore, this findings needs to be further confirmed by the experimental laboratory settings [61]. Genome‐wide association studies (GWASs) can be employed to reliably identify missense SNPs in patients causing diseases [60, 62]. In addition, In vitro and In vivo investigation of the oncogenicity of the human SSAT1 mutant (I21N) is necessary to strengthen the bioinformatics‐based findings in our current study [63, 64].

## 6 Conclusion

The present study identified rs757435207 (I21N) as the most deleterious and pathogenic missense SNP, which was found to be situated in the highly conserved residue and decreased the protein stability. MDS further unveiled adverse effects of the I21N variant causing significant deviation in the conformational dynamics and stability of the SSAT1 protein. Therefore, this study provides insight into the loss of enzymatic activity and functional interactions, which is crucial for understanding the I21N mutation‐mediated oncogenic behaviors. The results of this study would be helpful to filter the missense SNP for experimental identification of an oncogenic variant of the human *SAT1* gene.

## Author Contributions

**Md. Mozibullah:** conceptualization (equal), data curation (lead), formal analysis (lead), investigation (lead), methodology (lead), project administration (supporting), resources (lead), software (lead), supervision (supporting), validation (lead), visualization (lead), writing – original draft (lead), writing – review and editing (supporting). **Marina Khatun:** conceptualization (equal), methodology (supporting), project administration (lead), resources (supporting), supervision (lead), writing – review and editing (lead). **Md. Asaduzzaman Sikder:** project administration (supporting), supervision (supporting), writing – review and editing (equal). **Mohammod Johirul Islam:** project administration (supporting), supervision (supporting), writing – review and editing (supporting). **Mehbuba Sharmin:** data curation (supporting), investigation (supporting), validation (supporting), visualization (supporting).

## Conflicts of Interest

The authors declare no conflicts of interest.

## Supporting information


**Table S1.** All SNP that are deposited in the dbSNP database are enlisted and counted.
**Table S2.** Functional and pathogenicity prediction of all missense variants.
**Table S3.** The URL links of the used tools and servers in the study.

## Data Availability

All data generated or analyzed during this study are included in this article (and its supplementary information files).
